# Small subunit ribosomal metabarcoding reveals extraordinary trypanosomatid diversity in Brazilian bats

**DOI:** 10.1371/journal.pntd.0005790

**Published:** 2017-07-20

**Authors:** Maria Augusta Dario, Ricardo Moratelli, Philipp Schwabl, Ana Maria Jansen, Martin S. Llewellyn

**Affiliations:** 1 Laboratório de Biologia de Tripanosomatídeos, Instituto Oswaldo Cruz, Fundação Oswaldo Cruz, Rio de Janeiro, Rio de Janeiro, Brazil; 2 Fiocruz Mata Atlântica, Fundação Oswaldo Cruz, Rio de Janeiro, Rio de Janeiro, Brazil; 3 Institute of Biodiversity, Animal Health and Comparative Medicine, University of Glasgow, Glasgow, Scotland, United Kingdom; Instituto de Investigaciones Biotecnológicas, ARGENTINA

## Abstract

**Background:**

Bats are a highly successful, globally dispersed order of mammals that occupy a wide array of ecological niches. They are also intensely parasitized and implicated in multiple viral, bacterial and parasitic zoonoses. Trypanosomes are thought to be especially abundant and diverse in bats. In this study, we used 18S ribosomal RNA metabarcoding to probe bat trypanosome diversity in unprecedented detail.

**Methodology/Principal Findings:**

Total DNA was extracted from the blood of 90 bat individuals (17 species) captured along Atlantic Forest fragments of Espírito Santo state, southeast Brazil. 18S ribosomal RNA was amplified by standard and/or nested PCR, then deep sequenced to recover and identify Operational Taxonomic Units (OTUs) for phylogenetic analysis. Blood samples from 34 bat individuals (13 species) tested positive for infection by 18S rRNA amplification. Amplicon sequences clustered to 14 OTUs, of which five were identified as *Trypanosoma cruzi* I, *T*. *cruzi* III/V, *Trypanosoma cruzi marinkellei*, *Trypanosoma rangeli*, and *Trypanosoma dionisii*, and seven were identified as novel genotypes monophyletic to basal *T*. *cruzi* clade types of the New World. Another OTU was identified as a trypanosome like those found in reptiles. Surprisingly, the remaining OTU was identified as *Bodo saltans*–closest non-parasitic relative of the trypanosomatid order. While three blood samples featured just one OTU (*T*. *dionisii*), all others resolved as mixed infections of up to eight OTUs.

**Conclusions/Significance:**

This study demonstrates the utility of next-generation barcoding methods to screen parasite diversity in mammalian reservoir hosts. We exposed high rates of local bat parasitism by multiple trypanosome species, some known to cause fatal human disease, others non-pathogenic, novel or yet little understood. Our results highlight bats as a long-standing nexus among host-parasite interactions of multiple niches, sustained in part by opportunistic and incidental infections of consequence to evolutionary theory as much as to public health.

## Author summary

Bats make up a mega-diverse, intensely parasitized order of volant mammals whose unique behavioural and physiological adaptations promote infection by a vast array of microorganisms. Trypanosomes stand out as ancient protozoan parasites of bats. As cryptic morphology, low parasitaemia and selective growth in culture have recurrently biased survey, we used 18S ribosomal RNA metabarcoding to resolve bat trypanosomatid diversity in Atlantic Forest fragments of southeast Brazil. Next to several unknown species, our deep sequence-based detection and assignment protocol recognized multiple known human-pathogenic trypanosomes, another linked to reptile hosts as well as a non-parasitic kinetoplastid in the blood of various phyllostomid bats. The striking permissivity exposed here, in a region where bat trypanosomes recently featured in a fatal case of Chagas disease, compels further research on bats’ role in the dispersal and spill-over of various microorganisms among humans and wildlife.

## Introduction

*Trypanosoma cruzi* is the etiological agent of Chagas disease, a complex zoonosis that continues to take dozens of human lives each day [1]. Alongside its close relative *Trypanosoma cruzi marinkellei* in the *Schizotrypanum* subgenus, this important protozoan flagellate belongs to a broader, inter-continental group (the “*T*. *cruzi* clade”) of ancient endoparasites found to infect the mammalian fauna far and wide [2–3]. Infections have been reported in primates of Africa [4], marsupials of Australia [5] and a multitude of terrestrial mammals across the Americas [6], but most of this striking spread in host diversity tallies to few taxa within the clade (above all to *T*. *cruzi sensu stricto*, i.e., *T*. *cruzi*, and to *T*. *rangeli*).

The majority of *T*. *cruzi* clade diversity is found in bats. Chiroptera are known to carry both generalists such as *T*. *cruzi* and *T*. *rangeli* as well as multiple bat-restricted species—some abundant (e.g., *T*. *c*. *marinkellei*, *T*. *dionisii* and *T*. *erneyi*), others rare (e.g. *T*. *livingstonei* and *T*. *wauwau*) [3, 7–8]. Chiropteran immunity is unique with respect to other mammalian genera, coincident perhaps with physiological adaptations to flying [9]. Several features of bat immunity may predispose bats to long-term asymptomatic infections [10] with viruses [11–12], bacteria [13–14], fungi [15–16], protozoa [17–18] and helminths [19–20], several of which cause disease in humans and animals [21].

Given the diversity of bat-infecting *T*. *cruzi*-clade trypanosomes throughout the New and Old Worlds, many now accredit the Chiroptera with a fundamental role in the evolution of this parasite group [22]. In fact, the most parsimonious explanation to date for the origin and past expansion of the *T*. *cruzi* clade suggests a common ancestral lineage of bat-restricted trypanosomes that diversified into several independent lineages that on rare occasion switched into other terrestrial mammal hosts [17]. Bats’ recurrent interaction with other mammals and their various ectoparasites are thought to have afforded enough opportunity for at least five such switching or “seeding” events, likely since the early Eocene (54 to 48 million years ago) [7].

Many trypanosomes from bats are morphologically indistinguishable, often described simply as “*T*. *cruzi*-like” in the past [23]. As mixed species/genotype infections are probably common but overlooked or mistaken, molecular barcoding presents expedient recourse in resolving intricate trypanosomatid taxonomy and ecology. Metabarcoding couples classic molecular barcoding with next generation sequencing techniques [24–25] to generate thousands of sequence reads from a single sample [26–27]. These reads correspond to the diversity and abundance of organisms infecting the host individual [28–30].

In this study, we applied next-generation metabarcoding methods to the most bat-diverse (per area) biome of Brazil [31]. We focused on a degraded section of Atlantic Forest in Espírito Santo (ES) state where terrestrial mammals appear reduced in abundance as well as in *T*. *cruzi* infection. A fatal case of human *T*. *cruzi* (I-IV) and *T*. *dionisii* coinfection [32] immediately predated the bat trypanosome survey by 18S ribosomal RNA deep sequencing in this region.

## Methods

### Ethical statement

The sampling procedures reported herein were authorized by the Brazilian Institute of the Environment and Renewable Natural Resources (IBAMA) under license no. 19037–1 (23-05-2009). Euthanasia and blood collection met guidelines set by the Federal Council of Veterinary Medicine, Resolution 1000 (11-05-2012), in accordance to Federal Law 11.794/2008. All procedures followed protocols approved by the Oswaldo Cruz Foundation (Fiocruz) Ethics Committee for Animal Research (L0015-07).

### Study area, bat capture and sampling

Bat captures were carried out in two periods of 2015: June (dry season) and November (rainy season). Mist nets were opened upon sunset for four hours on two consecutive nights at each study location. A total of 108 bats were captured using ten mist nets (3 x 9 m, 35 mm mesh) placed along forest edges near banana and coffee crops at three different rural locations in Guarapari municipality, ES state, southeast Brazil: Rio da Prata (350 m a.s.l.), where a fatal case of Chagas disease occurred in 2012; Buenos Aires (250 m a.s.l.), where reports of triatomine invasion have increased in recent years; and Amarelos (at sea level), where triatomines have not been reported from the domestic zone (based on records by the Zoonosis Control Center, Guarapari municipality, ES) (S1 Fig).

Taxonomic identification by morphology followed [33] and a maximum of ten individuals per species (per site) were kept for further sampling, as specified by law. Once anesthetized with acepromazine (2%) in 9:1 ketamine hydrochloride (10%), these individuals were cleared of fur in the pectoral region (by scalpel) and sterilized with antiseptic soap and iodinated ethanol (70%) for blood withdrawal by cardiac puncture. Within the safety area of a flame, 300 μl blood was collected into sterile 1.5 ml vials and stabilized in two parts (i.e., 600 μl) 6 M Guanidine-HCl, 0.2 M EDTA solution for storage at -20°C. All bats used in these analyses received a collection number with the initials of the collector (RM) and were prepared for fluid preservation. This material will be subsequently deposited at the mammal collection of Museu Nacional, Federal University of Rio de Janeiro, Rio de Janeiro, Brazil.

### 18S rRNA amplification and deep sequencing

DNA was purified from 90 guanidine-EDTA blood lysates in DNeasy mini spin columns (Qiagen), with each of nine extraction rounds including one negative control. Purified DNA samples were then PCR-amplified with primers 5’-TGGGATAACAAAGGAGCA-3’ (forward) and 5’-CTGAGACTGTAACCTCAAAGC-3’ (reverse) for 30 cycles of 94°C (30 s), 55°C (60 s) and 72°C (90 s) to target a trypanosome-specific, ~556 bp region of the 18S rRNA gene as established in [5]. For a subset of samples, a wider, ~927 bp region (encompassing the ~556 bp above) was first targeted with external primers 5’-CAGAAACGAAACACGGGAG-3’ (forward) and 5’-CCTACTGGGCAGCTTGGA-3’ (reverse) at equivalent cycling conditions to form a nested (two-round) PCR amplification procedure following [34]. Sterile water (2x) and sample-free eluate from prior DNA purification (1x) were used to provide three negative controls per 20-sample PCR reaction. Amplicons were single-end barcoded [35], purified by agarose gel electrophoresis (PureLink Quick Gel Extraction Kit, Invitrogen), quantified by fluorometric assay (Qubit 2.0, Thermo Fisher Scientific) and pooled to equimolar concentration for multiplexed, paired-end (2 x 300 bp) sequencing on the Illumina MiSeq platform (Reagent Kit v2).

### Species delimitation and phylogenetic analysis

Amplicon sequences were filtered to retain only full-length reads of ≥ 99.9% base call accuracy by windowed trimming in Sickle [36], verified for quality in FastQC [37] and mapped against a *Trypanosoma* spp. reference collection from SILVA v119 [38] using Bowtie 2 [39]. Operational Taxonomic Unit (OTU) construction proceeded by UPARSE algorithm in USEARCH [40] and BLAST-based taxonomic assignment in the QIIME environment [41], with run parameters established during prior *in silico* testing on trypanosomatid 18S rRNA sequences from NCBI. Samples were clustered to OTUs *de novo* at 98% sequence similarity and assigned to extant species with a confidence threshold of 80%. Unassigned clusters were considered valid OTUs only if present at > 300 reads in any single sample and present at > 600 reads across all samples of the dataset.

Following OTU establishment, sequence read pairs from one representative per OTU were merged and aligned in Clustal W (with manual refinement of misplaced reads). Phylogenies were inferred in Mega 6 [42] by maximum likelihood (ML) tree construction under Kimura’s two-parameter model of nucleotide substitution with gamma-distributed variation among sites (K2 + G). One thousand bootstrap replicates were run to establish nodal support. The 50 18S rRNA reference sequences applied in phylogenetic analyses are listed with accession numbers in S1 Table. All sequences have been deposited in the NCBI Sequence Read Archive (SRA) under accession numbers SRR5451077-SRR5451120.

## Results

### Bat abundance and diversity

Of the 108 bats captured at Amarelos, Buenos Aires and Rio da Prata study sites, 105 individuals represent 16 species in the Phyllostomidae family, and three individuals represent one species (*Myotis nigricans*) in the Vespertilionidae family. Species and their abundances are listed in Table 1.

**Table 1:** Bat species captured in Guarapari municipality, ES state, Brazil.

Bat species	Capture sites		
	Amarelos	Buenos Aires	Rio da Prata
*Anoura geoffroyi*	-	-	3
*Anoura caudifer*	1	-	4
*Artibeus fimbriatus*	-	2	1
*Artibeus lituratus*	9	3	4
*Carollia perspicillata*	17	10	12
*Desmodus rotundus*	9	-	1
*Glossophaga soricina*	3	-	-
*Micronycteris* sp.	2	-	-
*Myotis nigricans*	2	1	-
*Phyllostomus discolor*	2	-	2
*Phyllostomus hastatus*	1	-	-
*Platyrrhinus lineatus*	-	-	2
*Platyrrhinus recifinus*	-	1	3
*Rhinophylla pumilio*	-	-	6
*Sturnira lilium*	2	1	2
*Tonatia bidens*	1	-	-
*Trachops cirrhosus*	1	-	-
**Total**	**50**	**18**	**40**

### Trypanosomatid abundance, diversity and distribution in bats

**Fig 1:**
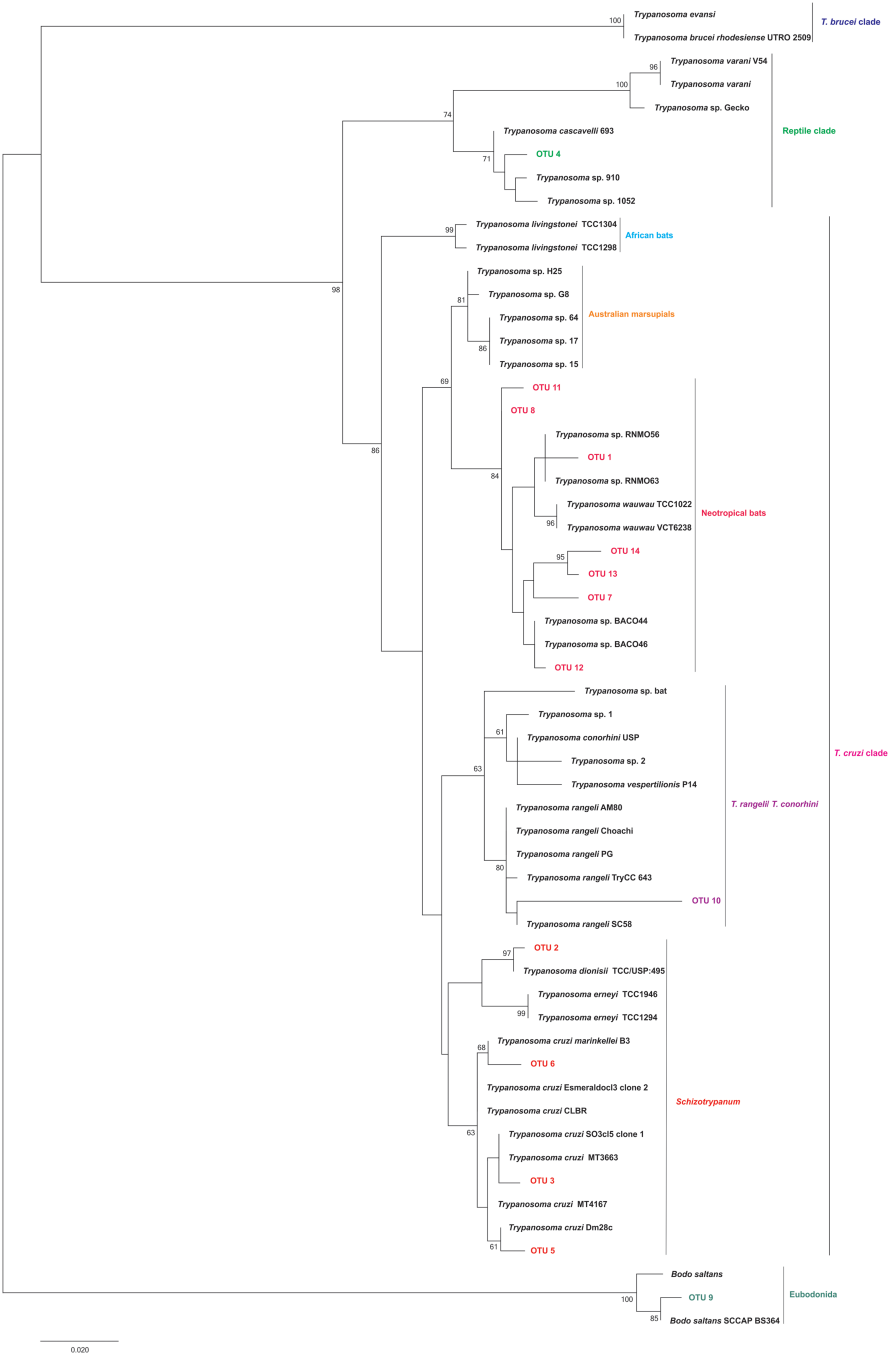
Phylogenetic placement of kinetoplastid OTUs detected in bats of Guarapari municipality, ES state, Brazil. Tree construction from 18S rRNA followed the maximum likelihood (ML) method under Kimura’s two-parameter model and gamma-distributed variation among sites (K2 + G). Numbers at nodes indicate support from 1000 bootstrap replicates. The 14 OTUs clustered into the *T*. *cruzi* clade (OTUs 1, 2, 3, 5, 6, 7, 8, 10, 11, 12, 13 and 14), a reptile-associated region (OTU 4) and the *B*. *saltans* outgroup (OTU 9).

**Fig 2:**
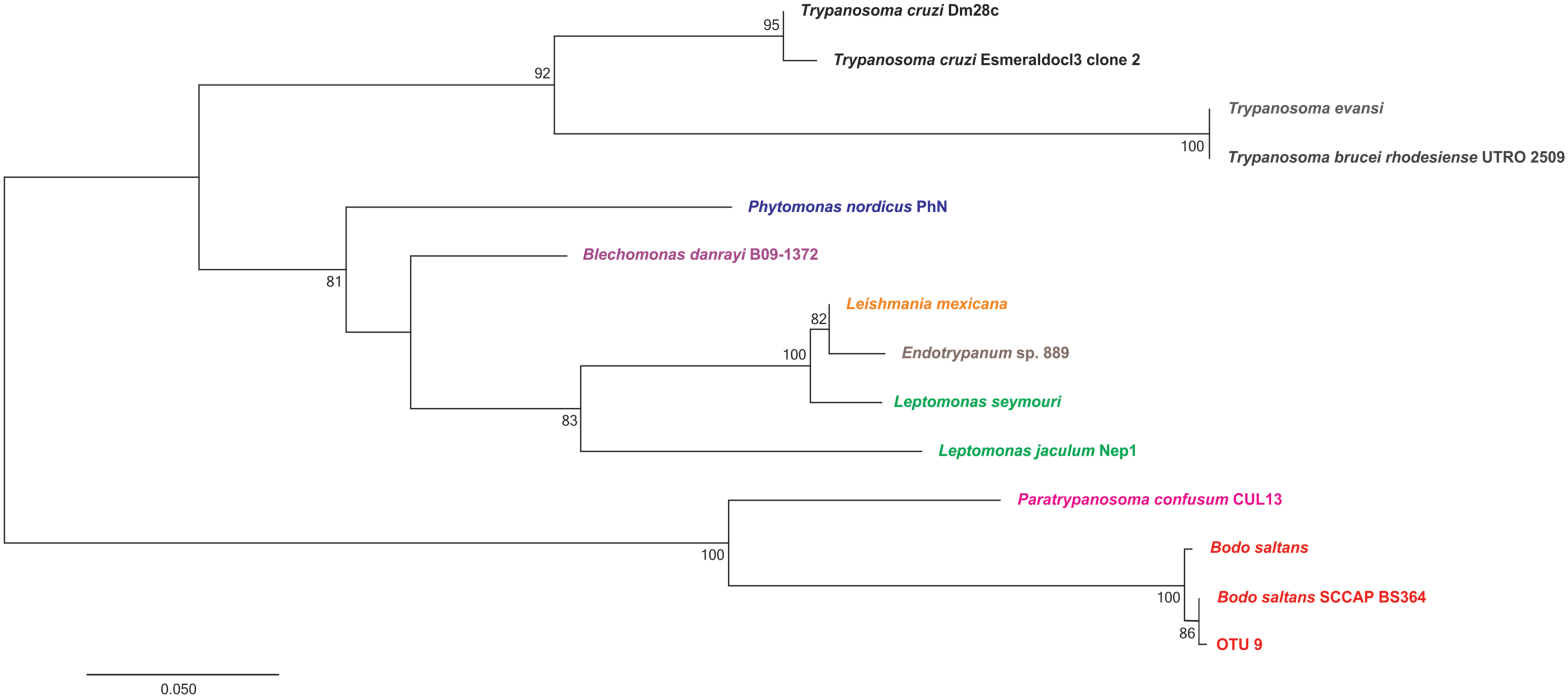
Phylogenetic placement of OTU 9 with *Bodo saltans* among a wider set of trypanosomatid genera. Tree construction from 18S rRNA followed the maximum likelihood (ML) method under Kimura’s two-parameter model and gamma-distributed variation among sites (K2 + G). Numbers at nodes indicate support from 1000 bootstrap replicates.

Standard and/or nested PCR amplified 18S rRNA gene fragments from 34 of 90 (38%) bat blood samples. The 34 positive samples derived from 13 bat species (of 17 species analysed) and comprised 14 distinct kinetoplastid OTUs. Five OTUs were assigned to *T*. *cruzi* I (OTU 3), *T*. *cruzi* III/IV (OTU 5), *T*. *c*. *marinkellei* (OTU 6), *T*. *rangeli* lineage D (OTU 10) and *T*. *dionisii* (OTU 2). A further seven OTUs did not assign to any known species of the *T*. *cruzi* clade. Phylogenetic analyses placed these seven OTUs (1, 7, 8, 11, 12, 13 and 14) within a monophyletic group that includes trypanosome species from bats of the New World. Finally, two OTUs showed greater homology outside of the *T*. *cruzi* clade—OTU 4, similar to a trypanosomatid species found in reptiles, and OTU 9, nearly identical to the eubodonid *Bodo saltans* (Figs 1 and 2, S1 Table).

**Fig 3:**
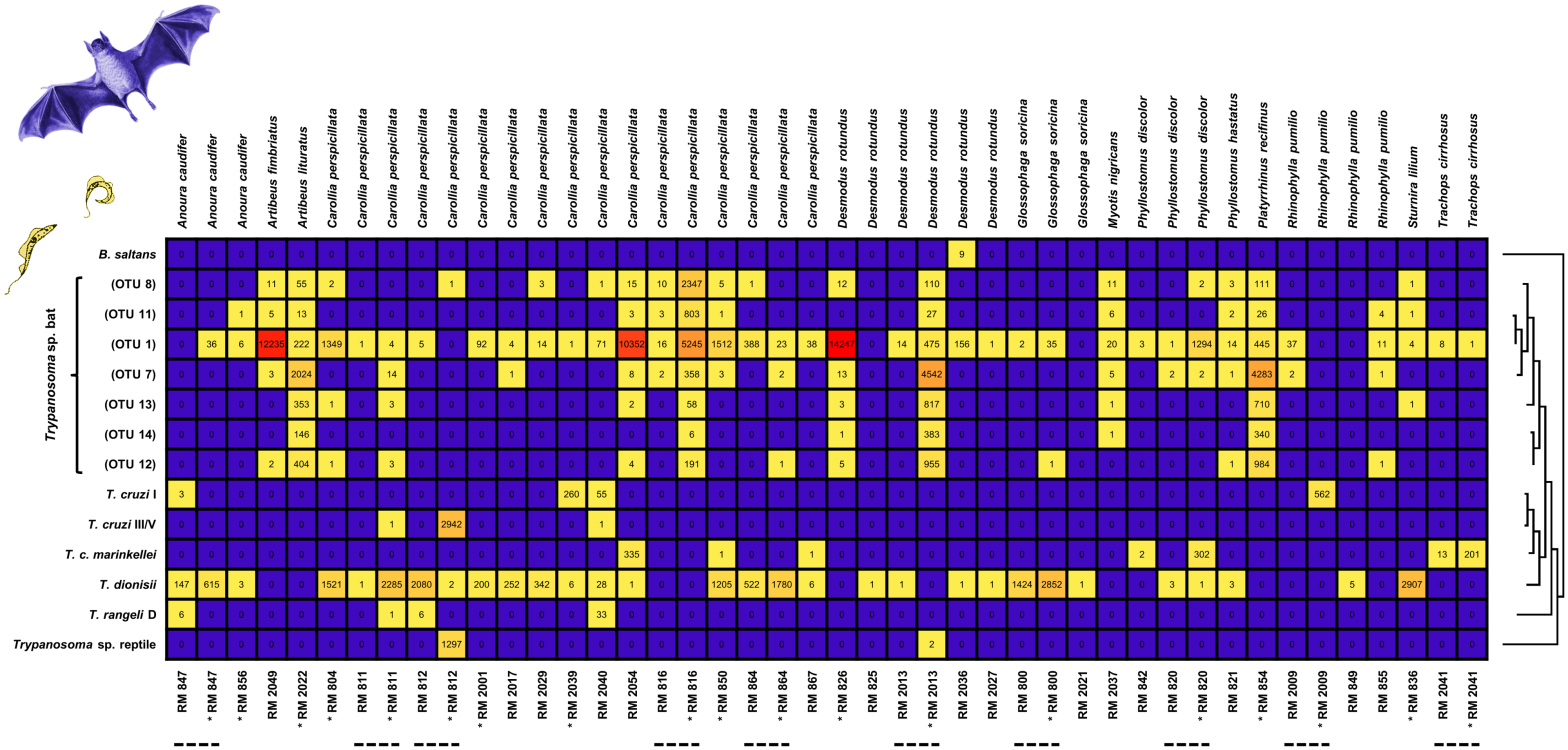
Heatmap of kinetoplastid OTU distribution among bats captured in Guarapari municipality, ES state, Brazil. Each column represents the infection profile of one infected bat individual. Cell colour denotes the sequence read intensity attributed to each OTU (left), increasing from purple (zero reads) through yellow into red. Bat species and sample IDs are given above/below. Asterisks indicate samples subjected to nested PCR, of which ten also underwent standard PCR (dashed lines). Phylogenetic relationships inferred from 18S rRNA by maximum likelihood (ML) tree construction are plotted at right.

**Fig 4:**
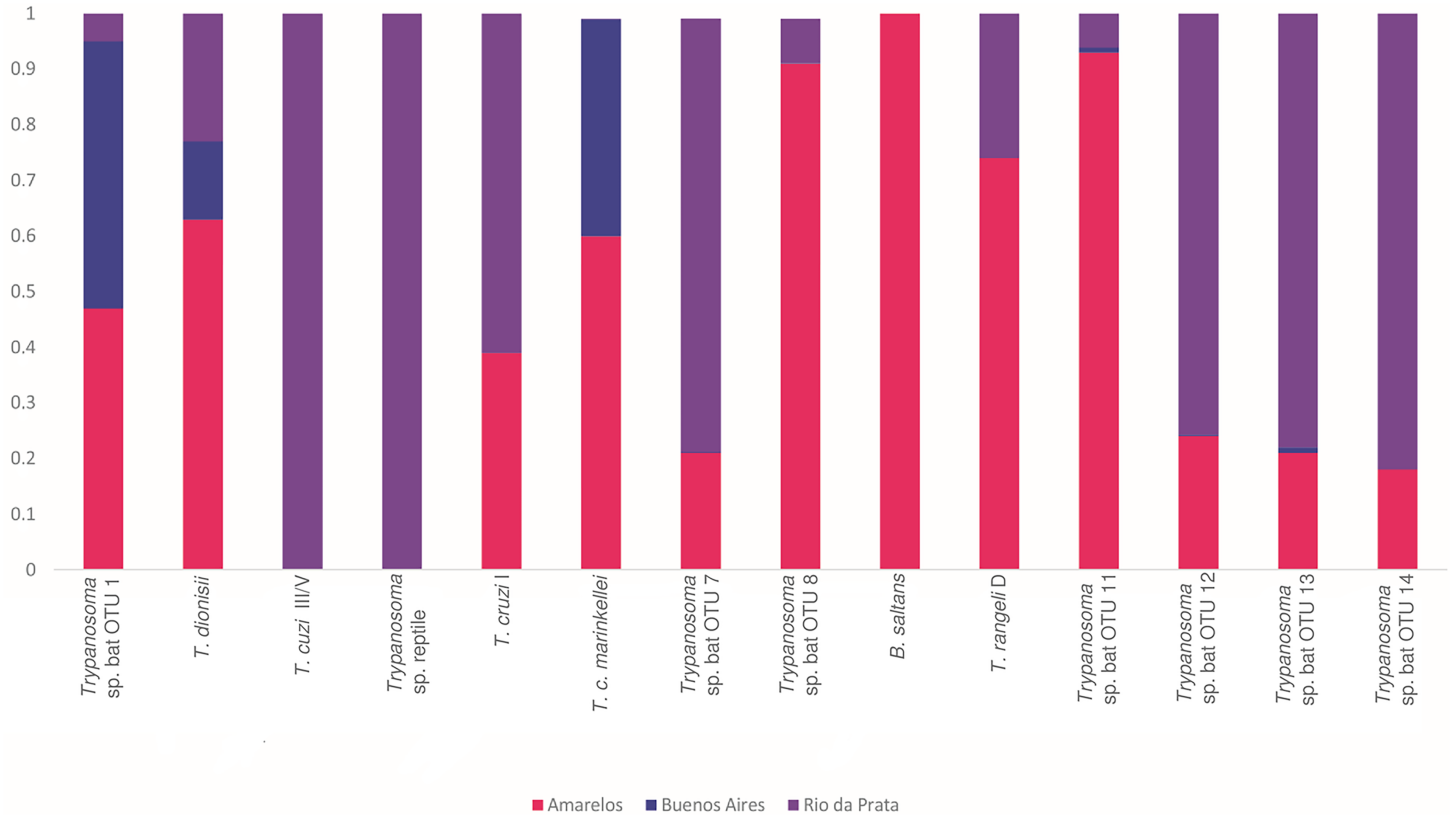
Kinetoplastid OTU distribution among study locations in Guarapari municipality, ES state, Brazil. Sequence reads attributed to each OTU are color-coded by proportions obtained from bats captured at Amarelos (magenta), Buenos Aires (blue) and Rio da Prata (violet) study sites.

Most trypanosome-infected bats presented mixed infections by two to eight OTUs. Only three positive blood samples (from *D*. *rotundus*, *G*. *soricina* and *R*. *pumilio*) contained a single OTU (*T*. *dionisii*; OTU 2). The bat species *A*. *lituratus*, *C*. *perspicillata*, *D*. *rotundus* and *P*. *recifinus* presented greatest trypanosome diversity, with seven to eight OTUs per species (Fig 3). Across the three study sites, trypanosomatid diversity and abundance broadly reflected bat capture success rather than any feature of the capture environment (Table 1 and Fig 4).

### Standard vs. nested PCR sensitivity

Nested PCR detected between one and six more OTUs than standard PCR in eight of ten samples subjected to both procedures, showing less sensitivity only in samples RM 847 and RM 2009—one and two less OTUs amplified, respectively (Fig 3).

## Discussion

In this study, we exposed unforeseen bat trypanosome 18S rRNA diversity from standard capture effort in Atlantic Forest fragments of Guarapari municipality, ES, southeast Brazil. Our metabarcoding approach identified a preponderance of coinfection, involving several human-pathogenic and bat-associated types of the *T*. *cruzi* clade, as well as a swathe of yet undescribed diversity closer to its base. Furthermore, we identified sequences from two divergent kinetoplastid taxa—one similar to trypanosomatid isolates from reptiles, another matching the non-parasitic *B*. *saltans*.

Unprecedented as they may be as complex co-infections, the diversity of individual kinetoplastids we report is not unexpected. Every recent trypanosome survey of bats has revealed novel parasite genotypes, host- and/or geographic range [8, 43–50], with particular surges in discovery following intensified sampling (e.g., transcontinental archival analysis) [8] or innovative approach (e.g., coalescent species delimitation) [43]. The 18S rRNA deep sequencing in bats here identifies further diversity around the most basal *T*. *cruzi* clade trypanosomes of the New World, with seven independent and novel taxonomic units forming sister groups to *T*. *wauwau* and *Neobat* species found in mormoopid and phyllostomid bats [8]. This expansion of a group related more closely to trypanosomatids detected in Australian marsupials than to those known from other neotropical mammals’ points to the Chiroptera as an ancient, perhaps original host order of the *T*. *cruzi* clade. Our data reinforce the bat host range of *T*. *cruzi*-clade trypanosomes across frugivorous, nectarivorous, carnivorous, generalist and hematophagous phyllostomid genera (*Anoura*, *Artibeus*, *Carollia*, *Desmodus*, *Glossophaga*, *Platyrrhinus*, *Phyllostomus*, *Rhinophylla*, *Sturnira*, *Trachops*) and into the (primarily insectivorous) Vespertilionidae.

Our study provides strong, if circumstantial, evidence for the role of bats as *T*. *cruzi* reservoirs in ES state. *Trypanosoma cruzi* I and III/V found in bats of this study correspond to Discrete Typing Units (DTUs) associated with a recent fatal *T*. *cruzi*–*T*. *dionisii* mixed infection and occur in *Triatoma vitticeps* at the study site [32]. These DTUs were not detected in parasitological or serological tests on local rodents and marsupials [32]. *Triatoma vitticeps* is thought to have poor stercocarian vector competence [51] and oral transmission via insectivory may be one of the few ways in which this species propagates disease. The apparent transfer of trypanosome diversity *en masse* from bat to human host via ingestion of the vector [32] supports transmission efficiency reported elsewhere in oral outbreaks [52]. Furthermore, given the low terrestrial mammal abundance in the heavily fragmented region where the samples were collected [32], bats may function here as principal reservoirs of parasites. There is growing evidence of bats’ potential in the maintenance of zoonotic *T*. *cruzi* transmission elsewhere in South America. For example, recent molecular surveys rank bats as top feeding sources of synanthropic *T*. *cruzi*-infected triatomines throughout Colombia, emphasize bats’ bridging of domestic and sylvatic transmission cycles in rural areas of Ecuador [45] (where non-volant hosts have shown limited infection [53–54]) and implicate bats as long-term refuges for parasites in areas subject to transmission interventions in Argentina [46]. Evidence of a new *T*. *cruzi* genotype associated with anthropogenic bats (TcBat) is also accumulating from around the continent [45, 55–58]. TcBat was not, however, observed in this study.

Here, we also provide first report of *T*. *rangeli* lineage D in bats, a strain initially isolated from *Phyllomys dasythrix* in southern Brazil [59]. As the ecogeographical structure of the *Rhodnius* spp. complex is thought to drive lineage divergence in *T*. *rangeli* [60–61], an efficiently transmitted salivarian parasite, our detection of lineage D further north and beyond the Rodentia serves well to confirm theory. Its putative vector *R*. *domesticus* [62] occurs throughout the Atlantic Forest, often in bromeliads [63] that rely on nectarivorous bats (e.g., the specialist flower-feeder *A*. *caudifer*) for pollination [64].

Whilst the expansion of the range of *T*. *rangeli* comes as little surprise, the presence of trypanosomes (OTU 4) with reptilian affinities in our study population is perhaps more intriguing. Nonetheless, bats and reptiles do commonly co-occur in an arboreal niche. Ecological host-fitting, involving opportunistic host switching mediated by vectors' feeding patterns within an ecological niche, is thought to be a prevailing mode of trypanosome evolution [65]. Reptilian trypanosomes are transmitted by sand fly vectors [65–66], with reports from Amazonia (*Viannamyia tuberculate* [67]) as well as central Brazil (*Evandromyia evandroi* [68]). Shared microhabitat use among bats, reptiles and sand flies potentiates spill-over of the parasite.

Most trypanosomatid diversity observed in this study was associated with complex mixed infections, a likely consequence of bats’ gregarious way of life. Tolerance of intracellular pathogens in the Chiroptera [21] suggests that multiple subclinical/asymptomatic infections may well accumulate in these hosts before triggering pathology linked to adaptive immune reactions in other non-volant mammals [69–72]. Frequent mixed infections, often coupled with low parasitaemia, have impeded bat trypanosome surveys in the past, both in genotyping from primary samples (e.g., low sensitivity in classic barcoding) [73] and on cultured cells (e.g., growth bias) [55, 61, 74]. The data presented here suggest that deep sequencing can resolve both infection identity and complexity.

Although our study demonstrates the power of the metabarcoding approach, several caveats are relevant. Sensitivity to contamination and errors from amplification and sequencing are of foremost concern [27, 75]. We employed a variety of cautionary measures during sample processing (e.g., flame-sterilized blood withdrawal, multiple negative DNA extraction/amplification controls) and in the bioinformatic phase: prior to taxonomic inference, we sent sequenced amplicons through a severe quality filter (99.9% base call accuracy), absorbed potential artefactual variance into broad 98% similarity clusters and rejected unassigned OTUs present at low to moderate depth (< 300/600 reads). Nevertheless, our study would have benefited from the inclusion of traditional methods (e.g., microscopy, ex and in vivo culture) for validation and follow-up. Based on subunit rRNA, OTU 9, isolated from a single bat (*D*. *rotundus*), was assigned to *B*. *saltans*, considered the closest free-living relative of the parasitic trypanosomatids. This observation joins others in unsettling assumptions about putatively free-living, yet seldom studied protist taxa. For example, 18S rRNA analysis (complemented by microscopy and serological testing) found an apparent case of babesiosis in China to involve erythrocytic colpodellids, the closest “free-living” relatives of the parasitic Apicomplexa [76].

Regrettably, our field-based study passed over visual and biochemical tests that could have established the occurrence and viability of OTU 9 in mammalian tissue and we hesitate to entirely rule out environmental contamination as its source. *Bodo saltans* belongs to the most widely adapted, physiologically tolerant zooflagellates on Earth [77]. It abounds in soil and water and can also spread in aerosolized forms. As such, this eubodonid may in rare cases happen upon sampling equipment as well as resist certain antiseptic measures taken in the field. On account of its exceptional halotolerance [78], for example, *B*. *saltans* may withstand some iodine-based disinfection (as do other protozoans—e.g., *Cryptosporidium* and *Giardia* [79]), though very unlikely as performed in this study (i.e., with ethanol). More importantly, however, OTU detection does not require a living organism, only its DNA. Severe contamination from the “dead” DNA of protist flagellates has indeed preoccupied past rRNA sequence analysis (e.g., see methods in [80]). In any case, we suggest additional (environmental) control samples (e.g., vials opened in the field, topical swabs around the site of cardiac puncture) and laboratory efforts that distinguish DNA from viable cells (e.g., separation of lysed and non-lysed cells, RNA/DNA comparisons) to help test for such possibilities in future research.

In this section of Atlantic Forest, where a rural Chagas disease fatality in all likelihood involved a bat-feeding triatomine [32], our deep sequencing study highlights the role of the Chiroptera as a reservoir for trypanosomiases. Furthermore, the unprecedented transfer of *T*. *dionisii* to a human from a bat, as well as the presence of reptile-infecting and putatively non-parasitic kinetoplastids in the same bat population, highlights the role of bats as keystone species in parasite spill-over events. Many questions remain on how the role of sylvatic hosts in pathogen dispersal varies in space and time, upon change to environment and at the evolutionary scale. Research into these intricacies of complex zoonosis will require much further innovation with high-sensitivity, high-throughput tools. We point to the power of next-generation metabarcoding strategies in studies of trypanosomatid ecology and evolution and strongly commend their future complementation with non-molecular methods.

## Supporting information

S1 Fig Representative map of bat capture locations in Atlantic Forest of Guarapari municipality, ES state, Brazil. (TIF)

S1 Table GenBank reference sequences used in phylogenetic analyses of kinetoplastid 18S rRNA. (DOCX)
