# Supplementary figures and images for: Small subunit ribosomal metabarcoding reveals extraordinary trypanosomatid diversity in Brazilian bats
Source: PLoS Negl Trop Dis. 2017 Jul 20;11(7):e0005790. doi: 10.1371/journal.pntd.0005790 (PMC5544246; doi:10.1371/journal.pntd.0005790)

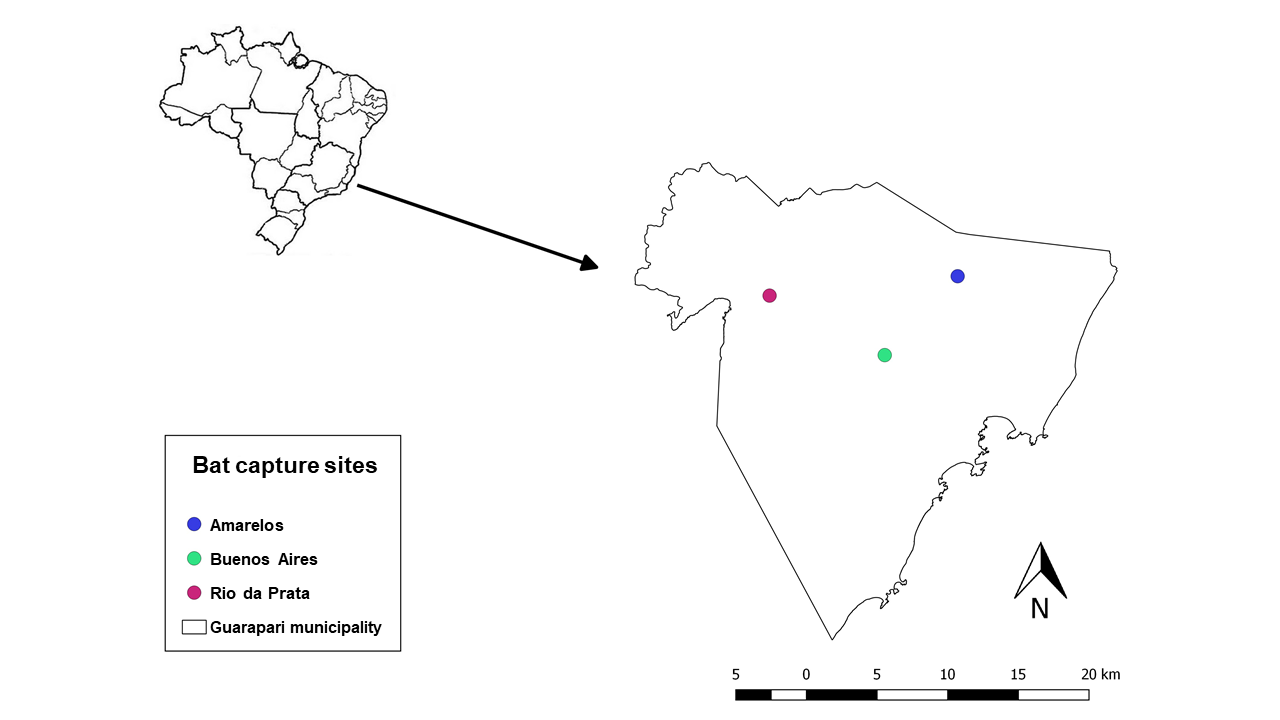

Supplement: S1 Fig — (TIF) [file pntd.0005790.s001.tif]
