# Supplementary material for: Small subunit ribosomal metabarcoding reveals extraordinary trypanosomatid diversity in Brazilian bats
Source: PLoS Negl Trop Dis. 2017 Jul 20;11(7):e0005790. doi: 10.1371/journal.pntd.0005790 (PMC5544246; doi:10.1371/journal.pntd.0005790)
Supplement: S1 Table — (DOCX) [file pntd.0005790.s002.docx]

**S1 Table: GenBank reference sequences used in phylogenetic analyses of trypanosomatid 18S rRNA.**

| **Isolate** | **Host Origin** | **GenBank Accession No.** |
| --- | --- | --- |
| *T. cruzi* Dm28c | *Didelphis marsupialis* | AF245382 |
| *T. cruzi* Esmeraldocl3 clone2 | *Homo sapiens* | AY785564 |
| *T. cruzi* MT3663 | *Panstrongylus geniculatus* | AF288660 |
| *T. cruzi* MT4167 | *Rhodnius brethesi* | AF288661 |
| *T. cruzi* SO3cl5 clone1 | *Triatoma infestans* | AY785579 |
| *T. cruzi* CLBR | *Triatoma infestans* | AF245383 |
| *T. c. marinkellei* B3 | Phyllostomus discolor | FJ649484 |
| *T. erneyi* TCC1294 | *T*adarida sp. | JN040988 |
| *T. erneyi* TCC1946 | *Mops condylurus* | JN040989 |
| *T. dionisii* TCC/USP495 | Carollia perspicillata | FJ0016667 |
| *T. rangeli* PG | *Homo sapiens* | AJ012417 |
| *T. rangeli* AM80 | *Homo sapiens* | AY491766 |
| *T. rangeli* Choachi | Rhodnius prolixus | AJ012414 |
| *T. rangeli* SC58 | Echimys dasythrix | AY491745 |
| *T. rangeli* TryCC643 | Platyrrinus lineatus | EU867803 |
| *Trypanosoma* sp. bat | *Rousettus aegyptiacus* | AJ012418 |
| *T. vespertilioni* P14 | *Pipistrellus pipistrellus* | AJ009166 |
| *Trypanosoma* sp. 2 | *Cercopithecus nictitans* | FM202493 |
| *T. conorhini* USP | *Rattus rattus* | AJ012411 |
| *Trypanosoma* sp. 1 | *Nandinia binotata* | FM2020492 |
| *Trypanosoma* sp. BACO44 | Artibeus lituratus | KT368797 |
| *Trypanosoma* sp. BACO46 | Artibeus lituratus | KT368798 |
| *T. wauwau* VCT6238 | Pteronotus gymnonotus | KT030840 |
| *T. wauwau* TCC1022 | Pteronotus parnellii | KT030830 |
| *Trypanosoma* sp. RNMO56 | Trachops cirrhosis | KT368795 |
| *Trypanosoma* sp. RNMO63 | Trachops cirrhosus | KT368796 |
| *Trypanosoma* sp. 64 | Trichosurus vulpecula | JN315383 |
| *Trypanosoma* sp. 17 | Trichosurus vulpecula | JN315382 |
| *Trypanosoma* sp. 15 | Trichosurus vulpecula | JN315381 |
| *Trypanosoma* sp. G8 | Bettongia penicillata | KC753537 |
| *Trypanosoma* sp. H25 | Macropus giganteus | AJ009168 |
| *T. livingstonei* TCC1298 | Rhinolophus landeri | KF192982 |
| *T. livingstonei* TCC1304 | Rhinolophus landeri | KF192983 |
| *Trypanosoma* sp. 1052 | Pseudoboa nigra | EU095839 |
| *T. cascavelli* 693 | *Crotalus durissus* | EU095845 |
| *Trypanosoma* sp. 910 | Viannamyia tuberculata | EU095838 |
| *Trypanosoma* sp. Gecko | Tarentola annularis | AJ620548 |
| *T. varani* | *Varanus exanthematicus* | AJ223572 |
| *T. varani* V54 | *Varanus exanthematicus* | AJ005279 |
| *T. evansi* | *Tabanus rubidus* | AY904050 |
| *T. brucei rhodesiense* UTRO 2509 | *Homo sapiens* | AJ009142 |
| *Leishmania Mexicana* | *Homo sapiens* | GQ332360 |
| *Endotrypanum* sp. 889 | *Psathyromyia dendrophyla* | EU021240 |
| *Leptomonas seymouri* | *Dysdercus suturellus* | AF153040 |
| *Leptomonas jaculum* Nep1 | *Nepa cinerea* | EF184218 |
| *Phytomonas nordicus* PhN | *Troilus luridus* | KT223609 |
| *Blechomonas danrayi* B09-1372 | Chaetopsylla globiceps | KF054137 |
| *Paratrypanosoma confusum* CUL13 | *Culex pipiens* | KF963538 |
| *Bodo saltans* SCCAP BS364 | n/a | AY998648 |
| *B. saltans* | n/a | JF693632 |
